# Communicating bad news in the practice of nursing: an integrative review

**DOI:** 10.31744/einstein_journal/2022RW6632

**Published:** 2022-07-21

**Authors:** Beatriz Lopes Agnese, Ana Carolina Queiroz Godoy Daniel, Rafaela Batista dos Santos Pedrosa

**Affiliations:** 1 Faculdade de Enfermagem Universidade Estadual de Campinas Campinas SP Brazil Faculdade de Enfermagem, Universidade Estadual de Campinas, Campinas, SP, Brazil.; 2 Hospital Israelita Albert Einstein São Paulo SP Brazil Hospital Israelita Albert Einstein, São Paulo, SP, Brazil.

**Keywords:** Health communication, Truth disclosure, Nurses, Education, nursing, Family, Attitude of health personnel

## Abstract

**Objective:**

To analyze current scientific knowledge about communication of bad news by nurses.

**Methods:**

This is an integrative literature review carried out by searching articles published in national and international journals indexed at SciELO, MEDLINE^®^ (PubMed^®^), Scopus, Bireme and CINAHL, from 2010 to 2020, by crossing the controlled descriptors “communication”, “revelation of the truth”, and “nursing”, and the uncontrolled descriptor “bad news”.

**Results:**

Ten articles with qualitative and cross-sectional design, as well as case reports were included. The analysis indicated the evidence available in the literature showed the nurses’ lack of ability to communicate bad news, although they are professionals who have close contact with patients and families and who establish a strong bond with them, and often face challenging situations for communicating bad news.

**Conclusion:**

There is an evident need to invest in training of nurses on skills to communicate bad news and establish a nurse-patient bond when dialoguing with the family. There are few studies in the literature addressing this issue; therefore, it is recommended to perform research that can contribute to improvements in the clinical practice and developing protocols to promote such care.

## INTRODUCTION

The process of transmitting and receiving ideas or knowledge is called communication, and is considered the fundamental element of human relationships and one of the most complex practices developed by humans. Through it, the individual recognizes themselves and sees their meaning before society, enabling the creation of bonds that shape not only them but everyone around as well.^(1,2)^

Communication is present in all human activities, whether through a look, a gesture, or a sentence. It is divided into two dimensions - verbal and non-verbal. The former occurs through words, which express an idea or thought, and the latter is characterized by gestures, features, tone of voice, and expression of emotions and feelings.^(1-3)^

The goal of communication is that it happens efficiently; it is necessary that there be a good understanding between both parties, which does not always happen, since there are many factors that influence it, such as expectations, culture, level of education, and values. It does not matter if the message is only transmitted, and, for this reason, it is necessary that the other party understands it.^(1-4)^

In the field of health, communication is the key element of the interactions, whether among the teams or with the team-patient/family. Thus, it is essential that these professionals have a good ability to communicate with their patients, favoring the bond team-patient/family, besides helping in assimilation of new realities and reduction of emotional impacts.^(5)^

The nurse is the health professional who is constantly in contact with patients and their families, whether in hospital wards or in primary healthcare units. It is through efficient communication that nurses can reduce stress and anxiety of those assisted, promoting quality care. Thus, it is essential that these professionals develop communication skills.^(3,6)^

The greatest challenge of communication between nurses and patients is bad news,^(7,8)^which consists of all pices of information given that drastically and negatively alters an individual’s life, changing their future perspective.^(9)^ This can happen by means of a definitive diagnosis, functional loss, painful treatment, prolonged hospitalization, and death. It is important to emphasize there are countless situations that qualify as bad news, and each one of them affects individuals in a particular way.^(8,9)^

The act of communicating bad news is often avoided and feared by the individuals who will receive it, and, they use several distancing strategies to postpone it.^(8)^ Feelings such as anguish, fear, discomfort, and even hostility - both from their ill loved one and from other family members - are present in the person who expects to receive bad news, and it is the nurse’s duty to share information in an efficient and welcoming manner. For this reason, they require technical qualification and constant training.^(8,10)^

Currently, communication of bad news is a little studied theme, especially in the field of nursing.^(8,11)^ It is known that there are some methods and protocols to help in the process, but even so, there are particularities that should be adopted for each case, since the patient is a multifactorial being (psychological, social, and physical), with varying needs.^(10)^

Communication of bad news is not something instantly learned, but a skill to be developed and worked on during the entire working life of health professionals.^(3,6,7,11)^ The analysis of evidence on this nursing practice, by means of an integrative literature review, enables accumulating knowledge on the subject, and contributes to the identification and evaluation of effective communication strategies and development of professional training protocols, which allow patient-centered care and assistance with quality and dignity.

## OBJECTIVE

To analyze current scientific knowledge about communication of bad news by nurses.

## METHODS

This is an integrative review study, consisting of analysis, grouping, and synthesis of scientific evidence relevant to the desired subject, involving both theoretical and empirical literature, helping to form the study of a certain phenomenon, and generating knowledge to be used in quality clinical practice.^(12)^

This review adopted the methodological steps proposed by Mendes et al., as follows:^(13)^ identification of the research question that is relevant in the field of health and nursing for preparation of the integrative review; establishment of criteria for inclusion and exclusion of studies, sampling, or literature search; definition of pieces of information to be extracted from the selected studies/categorization of the studies; evaluation of the studies included in the integrative review; and interpretation of the results and presentation of the review and synthesis of knowledge.

The elements of the strategy PICO^(14)^ (P for patient or problem, I for intervention, C for comparison, and O for outcomes) were used to formulate the following guiding question, “What is the nurse’s knowledge about communicating bad news?”.

The search in scientific literature was conducted in 2020, in national and international journals indexed at Scientific Electronic Library Online (SciELO), Medical Literature and Retrieval System Online (MEDLINE^®^) via PubMed^®^, SciVerse Scopus (Scopus), Latin American and Caribbean Center on Health Sciences Information (Bireme), and Cumulative Index to Nursing and Allied Health Literature (CINAHL), via the Coordination for Improvement of Higher Education Personnel (CAPES - *Coordenação de Aperfeiçoamento de Pessoal de Nível Superior*) portal.

The descriptors used in the search are contained in the structured and multilingual vocabulary of the Medical Subject Headings (MeSH) and in the Health Sciences Descriptors (DeCS - *Descritores em Ciências da Saúde*). As an uncontrolled descriptor, the term “bad news” was included to broaden the results obtained. The search strategy in MEDLINE^®^ and Scopus included the combination of keywords: “truth disclosure” [All Fields] OR “communication” [All Fields] AND “nursing” [MeSH Terms]. For Bireme and SciELO, we used “truth disclosure” OR *“revelación de la verdad”* OR *“revelação da verdade”* [subject descriptor] OR “bad news” OR *“má notícia”* OR *“malas noticias”* [words] AND (“communication” OR *“communicación”* OR *“comunicação”)* [subject descriptor] AND (“nursing” OR *“enfermagem”* OR *“enfermeria”)* [subject descriptor]. Finally, for CINAHL: “truth disclosure” OR “disclosures, truth” OR “truth disclosures” OR “bad news” AND “communication” AND “nursing” were used.

The following criteria were adopted for selection of articles: all categories, regardless of the type of study conducted, whether primary or secondary characteristics (quantitative, qualitative, literature review, descriptive study, cross-sectional study, reflection study, clinical case report, among others); articles with abstracts and full texts available electronically; those published in Portuguese, English, or Spanish, between 2010 and 2020, and articles that indicated the topic of interest of this review in the title, abstract, or body of the text. Articles that did not meet the proposed theme for the study and those different from the proposed languages were excluded.

**Figure 1:**
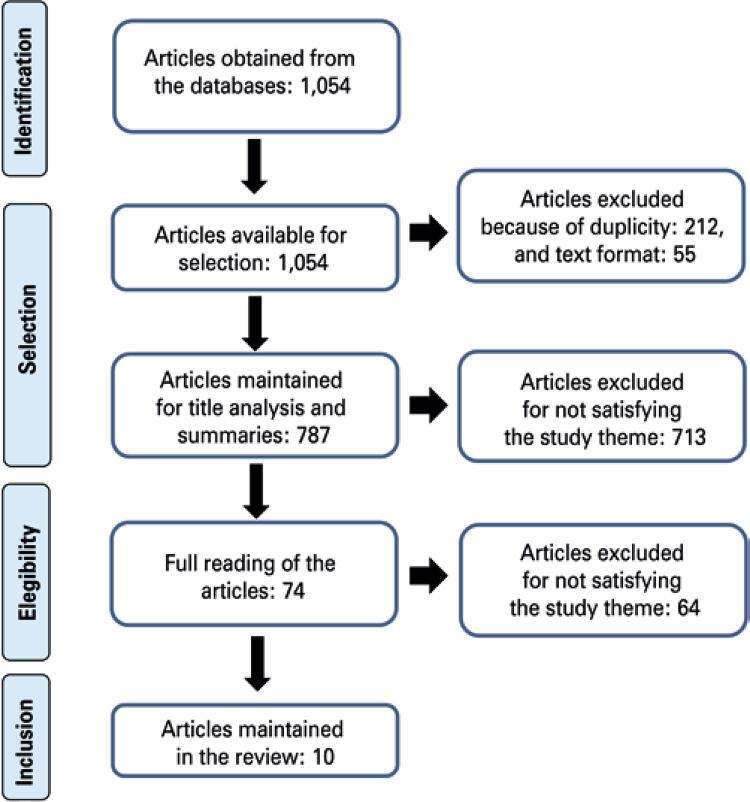
Flowchart of study search and selection

All articles included in the sample were exported to the software EndNote Web Basic (Clarivate Analytics^®^), and duplicate articles were removed. At first, the titles and abstracts of the total sample were read by two independent reviewers, taking into account the inclusion and exclusion criteria. Subsequently, the articles were read in full, selecting only those that met the study criteria. Figure 1 shows the flowchart of the search and selection of studies, according to the model of the Preferred Reporting Items for Systematic Reviews and Meta-Analyses (PRISMA).^(15)^

The instrument proposed by Ursi et al.^(16)^ was used in this study for data extraction, with the purpose of collecting and organizing the following information: name of the authors, name of the journal, year of publication, country of the study, language, name of the article, type of the study, sample, results (nurse’s abilities to communicate bad news, education, training, and previous experiences), method of data analysis, conclusion, recommendations, and quality assessment.

The evidence classification hierarchy proposed by Melnyk et al.,^(17)^ was used to describe the level of evidence of the studies: level I for systematic reviews or meta-analysis; level II for randomized controlled trial; level III for non-randomized clinical trials; level IV for cohort and case-control studies; level V for systematic review of descriptive and qualitative studies; level VI for descriptive or qualitative study, and level VII for opinion of authorities or expert committees.

## RESULTS

By combining the controlled descriptors and the uncontrolled descriptor “bad news”, a total of 1,054 articles were found, 69 in MEDLINE^®^, 177 in the Virtual Health Library*/*Bireme, eight in SciELO, 448 in Scopus, and 352 in CINAHL. Most of the articles were neither related to nor specifically reported on the subject of communication of bad news in relation to nurses and their role. Thus, only ten met the study criteria; they are presented on table 1.^(8,11,18,19-25)^

**Table 1:** Data collected from the articles selected for the review

Authors	Country of the study	Language	Study type	Sample	Results	Data analysis method	Conclusion and recommendations	Quality evaluation^*^
Warnock^(8)^	England	English	Case study	A patient with a chronic disease who needs to receive bad news	Nurses are responsible for a range of releases of bad news. The study shows difficulties and facilitators for good communication. It also shows that nurses are so involved in the process of communicating bad news that it ends up strengthening their relationship with their patients and families, benefiting the nurse-patient relationship	Analytical analysis of the data presented in the case and exploratory analysis of literature review to show the best techniques for communicating bad news	It is important to recognize nurses in the process of delivering bad news, and it is essential to prepare, guide, and train their communication skills	VII
Rocha et al.^(11)^	Brazil	Portuguese	Reflective study	Studies published in recent years, searched in a broad and non-systematic way, to foster debate on the subject and bring insight as to the topic of communicating bad news	The study places communication of bad news as a role of nurses as much as of physicians. To convey effective news, it must be given gradually and clearly, in conversation with the team, observing the emotional aspects of the patient and family members, and offering attentive and humanized listening. The authors also emphasize that there is no universal technique that works for everyone. Thus, it is necessary to act according to the ethical principles of justice, autonomy, beneficence, and non-maleficence	Reflective data analysis after literature review	Communicating bad news involves all professionals associated with the patient. In the obstetric scenario, it mainly involves the mother and family members. It is important to emphasize that in humanized and harmonious communication, it is necessary to pay attention to verbal and non-verbal communication. Moreover, due to the lack of studies in the literature on the communication of bad news by nurses, organizing training courses to develop such skills is suggested	VII
Amorim et al.^(18)^	Brazil	Portuguese	Qualitative, descriptive, and exploratory study	15 nurses from 10 Primary Healthcare Units located in the South Region of Brazil	According to the nurses who participated in the study, the barriers found in communicating bad news were related to service demand, work organization, user characteristics, and personal aspects. The advantages pointed to privacy and the fact of being in the community. Some points fit both in the barriers and facilitating communication: operation of the network, the team, professional training, professional experience, and personal aspects	Discourse analysis	There is scarce approach to the subject of “communicating bad news” during professional training. This creates a series of barriers for professionals. It is important that the subject is taken to educational organizations, for a discussion of the subject and the development of courses/materials on communication	VI
Corey et al.^(19)^	United States	English	Qualitative, exploratory, and descriptive study	5 nursing professionals, with 2 to 10 years of experience, from 2 working groups (Central Florida Chapter of the Oncology Nursing Society e Greater Orlando Hematology Oncology Physician Extenders)	The experiences of nurses in communicating bad news are based on their own communication skills. Although not appearing in several studies as the deliverer of bad news, nurses are professionals who experience this practice in their daily lives. Thus, it is necessary to develop their skills, especially in training environments. The use of the SPIKES protocol outside the context of oncology is seen as advantageous for those who want to start their studies in the field and put into practice the communication of bad news in a proper way	Extraction of common themes, from accounts, validation, and reliability by the triangulation method, concept verification, and grouping	It is essential to promote the development of communication skills in healthcare professionals, especially nurses, and to ensure emotional support to patients. In addition, the use of protocols for communicating bad news is important to assist in the development of such skills. The study shows a good use of the SPIKES protocol, and the interviewed nurses indicated they would continue with the use of SPIKES and recommend it to fellow professionals	VI
Imanipour et al.^(20)^	Iran	English	Descriptive and cross-sectional study	160 nurses from the intensive care units of hospitals affiliated with the Faculty of Medical Sciences of the University of Tehran	Nurses, especially from critical care, are directly involved in communicating bad news by playing different roles, providing emotional support to the patient and family, clarifying doubts and medical words in simple language, and preparing the patient and family to receive the bad news. “In this study, 78.8% of nurses had moderate knowledge on how to communicate bad news and few had a good level of knowledge (16.2%)”	Statistical analysis of the participants’ answers using the Statistical Package for the Social Science, version 2.0, and clustering of the main answers	Although the study shows the important role of nurses in communication of bad news and their positive attitude in the process, an inadequate level of knowledge required for good communication is demonstrated. Thus, it is necessary to create educational programs to improve the nurses’ communication skills	VI
Warnock et al.^(21)^	England	English	Descriptive and cross-sectional study	236 nursing professionals, including nursing coordinators, lecturers, head nurses, staff nurses, specialists, and midwives in National Health Service teaching hospitals, in the United Kingdom	Nurses participated in breaking bad news in all types of care. They proved to be unprepared, many times, by being caught off guard in having to deliver bad news, and by not having adequate training that would enable them to disclose bad news effectively. In addition, it is important to carry out the process of team communication. Nurses are often called by the patients themselves for clarification of misunderstood terms said by the doctors	Descriptive statistics from the coding of answers to the questionnaires, as per their contents and subsequent grouping by similar themes. The information was crossed, generating themes and categories. Finally, the codes, themes, and categories were compared, generating the final interpretation	“Guidance on breaking bad news must encompass the entire process of doing so and recognizing the challenges nurses face in the inpatient clinical field. Developments in education and support are needed that reflect the challenges nurses face in the inpatient care setting”	VI
Warnock et al.^(22)^	England	English	Descriptive and cross-sectional study	145 participants including nurses and other health professionals, working in the medical field in a region of northern England	The study provides a list of several difficulties presented by nurses of the service, at the time or in the process of breaking bad news. “Traditional issues, such as information on diagnosis and treatment, were described, but additional topics were identified, such as the impact of illness and end-of-life care.” A description was made of the issues most present as difficulties at the time of disclosure; these were: organization, the situation in which the disclosure is made, reactions and issues related to the patient and their families; and finally, personal issues, such as lack of skills or confidence	Structural analysis of the obtained data, with independent and collaborative phases, which identified some preliminary thematic indexes. Then, the similarities and differences were evaluated forming the final thematic index	The study provides “an understanding of the scope of challenges faced by staff”, when they are involved in the process of breaking bad news. With this, it is possible to see the importance of developing courses that improve the communication skills of professionals, especially in a way that focuses on their clinical practice	VI
Hemming^(23)^	England	English	Case study	A patient with a chronic disease, who has experienced several situations of receiving bad news from staff	Nurses play a central role in communicating bad news because they are in direct contact with the patient for longer than other professionals. In addition, establishing bonds makes them listen better to their patients and inform them of the news, according to their concerns, needs, and understanding. Also, good communication requires teamwork. Training in advanced communication skills has proven useful in the practice of conveying bad news	Exploratory and analytical analysis, based on current literature data, compared with the presented report	Specialist nurses proved to be very valuable in communicating bad news, mainly because of trust that is created between them and their patients/family members. Thus, the communication of bad news affects patients in different ways, educating them about the subject and reinforcing the idea of the need for more organizational investments in developing communication skills of its professionals	VII
Rosenzweig^(24)^	United States	English	Reflective study	Extensive use of literature data, and of the SPIKES protocol	Patient-centered communication ensures a better quality of care. Thus, when communicating bad news, nurses must maintain a plan to avoid errors in the process and ensure a good understanding in the communication, always based on empathy. The study shows the benefits of the SPIKES protocol for those who are at the beginning of their learning process regarding communication of bad news	Qualitative data analysis, comparing the use of the protocol with other communication data, and analyzing the existing content on the topic	The ability to communicate bad news is something that can be learned through communication development courses. These can and should be included in educational organizations around the world. Properly communicating bad news and using a protocol promotes a more comfortable process for nurses and their patients. This improves the overall communication among nurses and patients/family members	VII
Uveges et al.^(25)^	United States	English	Case study	1 nurse and 1 patient in a case report on the ethical aspect of the professional in communicating or not communicating bad news to patients and families	Nurses who work in intensive care units constantly participate in the delivery of bad news, either by clarifying technical terms, attending multidisciplinary meetings, assisting in the understanding of difficult news, and helping to discern the implications of the disease/condition. In addition, some patients and families prefer bedside conversations rather than meetings with the team. The study shows a nurse’s unpreparedness when faced with the ethical act of communicating, or not, bad news	Analytical analysis of the events that occured were describe in the report and compared to what the literature brings as best practice	Nurses are participants in the process of communicating bad news, which is not a one-time process, but one that occurs throughout the course of care. They act as educators, facilitators, and supporters of the patient and their family members during the communication. Therefore, the process must be done within ethical principles and the patient’s preferences. Their skills must be developed for best practice	VII

^*^ Levels of evidence I to VII^.(17)^

The analysis of the studies included in this review allowed us to identify three main points addressed in the articles. The first is about the little or no communication skills developed during nurses’ training, a situation described in all articles analyzed. The second point discussed is about how the disclosure of bad news appears during nurses’ practice, from primary to tertiary care.^(8,11,19,21,25)^ Finally, another aspect identified is the creation of the link between patient/family/nurse for an effective communication of bad news,^(22,23)^ as well as for strengthening the trust between them in difficult moments.^(8,18-25)^

Regarding the communication skills of students and nursing professionals in general, the studies showed that, during undergraduate course or technical training, there are no subjects or courses addressing this issue, and, consequently, this produces a deficit in the development of professional skills.^(20,21)^

The results of studies^(21,23,25)^point out that nurses, among the other health disciplines, are the professionals who have the largest workload of direct care delivery to patients and their families. This means that, regardless of the environment, whether in the inpatients´unit, intensive care unit, outpatient clinic, or primary healthcare unit, nurses are present at the moment of conveying bad news, as the agent of disclosure or a member of the interdisciplinary team.^(21,23)^

Regarding the nurse-patient bond, the results of studies^(8,22,23)^ showed the existence of the bond facilitates communication of bad news and strengthens ties.^(8,20,21)^

## DISCUSSION

Evidence proves communication of bad news by nurses is a subject still poorly explored.^(10,11)^ According to the findings of this study, it is possible to verify there is a discrepancy in the background of nurses regarding this theme, whether in undergraduate teaching or in the different work organizations that do not promote professional training. Studies show that there is a growing concern with quality communication, but investments are insufficient.^(22,26)^

The literature points out that there are few organizations that use protocols to help professionals at the time of announcing bad news.^(10)^The SPIKES tool^(27)^ is a medical protocol that contemplates six steps for the action in a didactic way, and has been used by nurses around the world to train their communication skills.^(10)^Two of the studies included in this review cite the benefits of using the SPIKES protocol, as well as the advantages of other protocols in clinical practice.^(19,24)^

Evidence shows the SPIKES protocol is a widely disseminated toll used in hospitals around the world, with free access and easy retrieval by health professionals.^(27-29)^It may be an ally for those who are beginning to learn the disclosure of bad news or feel insecure to perform this action, since the tool directs the conversation between professional-patient/family,^(29)^ and builds nurses’ communication skills.^(10)^

Since nurses have the largest workload and more contact with patients and family members, they are key players in the disclosure of bad news, and should establish an important bond at this moment.^(30,31)^ Results of previous studies showed the creation of the nurse/patient bond is essential for a relationship of trust between them.^(29,31)^ Therefore, even if it is not the nurses who communicate the bad news, their presence is fundamental to establish the necessary confidence to the patient and family, besides being the professionals who provide continuity of care to these subjects, and follows all consequences of the announced news.^(29,31)^

Among the studies selected, some of them demonstrated the nurses remain close to the patients and their family even after disclosure, clarifying terms, helping to understand the extent of the news, comforting the patient and family, and offering support.^(8,11,25)^ Therefore, it is important to highlight that this communication must occur as a team, because even if it is the physician or the nurse who conveys bad news, there is a repercussion that reaches all other professionals who deal with the patient.^(30)^

In communicating bad news in delivering comprehensive care, nurses are fundamental professionals who favor the establishment of a relationship of trust between the parties. This highlights the need to promote the development of communication skills of such professionals to ensure quality care.

This study had as limitations a reduced number of studies available on the subject, low levels of evidence in all studies found (with descriptive and qualitative designs and opinions of authorities or expert committees), exclusion of articles with no texts available on the CAPES portal, and exclusion of those published in languages other than Portuguese, English, and Spanish. The search did not find review studies that allowed comparisons with the findings of this research, demonstrating the need to explore the content in search of evidence that can promote the communication of bad news in clinical practice. Thus, this study provides a consolidation of information already published in the literature over the last 10 years.

## CONCLUSION

Nurses are frontline professionals in disclosure of bad news, but the deficiencies in their education and training are clear, culminating in inability to perform such a practice. Therefore, it is urgent to implement efforts for the training of communication skills in educational and work organizations. Moreover, the production of knowledge on this subject is also scarce, and it is recommended that new studies be carried out to add to and expand the discussion on the disclosure of bad news by nurses and, consequently, to enhance the quality of care for patients and their families.
